# Performance of *Trichogramma pretiosum* Riley (Hymenoptera: Trichogrammatidae) on eggs of *Helicoverpa armigera* (Hübner) (Lepidoptera: Noctuidae)

**DOI:** 10.1038/s41598-018-37797-9

**Published:** 2019-02-04

**Authors:** Valéria Lucas Laurentis, Dagmara Gomes Ramalho, Nathália Alves Santos, Vanessa Fabíola Pereira Carvalho, Alessandra Marieli Vacari, Sergio Antonio De Bortoli, Rodrigo Cassio Sola Veneziani, Gabriel da Costa Inácio, Bruno Gomes Dami

**Affiliations:** 1 Laboratory of Biology and Insect Rearing (LBIR), Department of Plant Protection, São Paulo State University FCAV-Unesp, 14884-900, Via de Acesso Prof. Paulo Donato Castellane s/n, Jaboticabal, São Paulo Brazil; 2 Department of Biology, São Paulo University FFCLRP-USP, 14040-900, Avenida Bandeirantes, 3900 Ribeirão Preto, São Paulo Brazil; 3 Animal Science Graduate Program, University of Franca Unifran, 14404-600, Avenida Dr Armando Salles de Oliveira, 201, Parque Universitário, Franca, São Paulo Brazil

## Abstract

*Helicoverpa armigera* (Hübner) (Lepidoptera: Noctuidae) is a polyphagous pest with a wide geographic distribution. This pest first arrived in Brazil in 2013, and since then studies on possible control methods for it have been necessary. A possible method for the control of *H*. *armigera* is using the egg parasitoid *Trichogramma pretiosum* Riley (Hymenoptera: Trichogrammatidae). Therefore, the objective of this study was to evaluate the performance of *T*. *pretiosum* on *H*. *armigera* eggs, which are known to represent suitable hosts for the development of this parasitoid species in the laboratory. Parasitism and emergence rates and the duration of the egg-to-adult period of *T*. *pretiosum* were investigated following 24- and 48-h exposures of this parasitoid to *H*. *armigera* and *Corcyra cephalonica* (Stainton) (Lepidoptera: Pyralidae) eggs. The longevity of offspring after the 24-h exposure was studied, as well as the frequency of parasitism and emergence, host preference, and the emergence of offspring from eggs of different ages or oviposited by lepidopterans on different days. Parasitism was 14.4 and 34.9% more frequent on *C*. *cephalonica* than on *H*. *armigera* after 24 and 48 h of exposure, respectively. In *C*. *cephalonica*, parasitism was 27.2% higher after 48 h. Parasitism was more frequent on *C*. *cephalonica* eggs collected on the second day of oviposition (76.2%), and on *H*. *armigera* on the third day of oviposition (71.1%). Parasitism frequency was lower on 2-day-old *C*. *cephalonica* eggs (63.3%) and on 3-day-old *H*. *armigera* eggs (41.3%). When tested with a chance of choice between hosts, *T*. *pretiosum* preferred *H*. *armigera*, while in the test with no chance of choice there was no difference in preference. Thus, *T*. *pretiosum* may be considered a tool for the Integrated Pest Management (IPM) of *H*. *armigera*.

## Introduction

*Helicoverpa armigera* (Hübner) (Lepidoptera: Noctuidae) is an important agricultural pest worldwide because it is highly polyphagous and damages several crops of economic importance, such as tomato, cotton, soybean, corn, and sunflower^1–4^. This species was first reported in Brazil in 2013 at high population sizes on soybean, corn, and cotton crops^5,6^. During its occurrence, farmers lost an estimated R$ 10 billion^7^.

The egg parasitoid *Trichogramma* spp. Westwood (Hymenoptera: Trichogrammatidae) is used in several countries for the control of lepidopteran pests^8–10^ by inundative application on millions of hectares of economically important agricultural crops^8,9,11,12^.

To meet demands for the biological control of pests of such crops as maize, soybean, cotton, tomato, and sugarcane some companies perform the mass-rearing of these parasitoids, and in Brazil four biofactories that produce *Trichogramma* spp. are registered in the Phytosanitary Agrochemicals System^13^. Among the species in the genus *Trichogramma*, *T*. *pretiosum* Riley (Hymenoptera: Trichogrammatidae) has been commercialized for the control of lepidopteran eggs^13–15^, and has been reported in approximately 18 different hosts and in 13 crops^16^.

In this context, *T*. *pretiosum* can be considered one of the tools available for use in the *H*. *armigera* Integrated Pest Management (IPM) program. However, to obtain good results using this parasitoid in the field, basic studies are necessary to better understand its development and behavior in the eggs of this pest. Therefore, the objective of the present study was to investigate the parasitism behavior of *T*. *pretiosum* on *H*. *armigera* eggs, compared with that on *Corcyra cephalonica* (Stainton) (Lepidoptera: Pyralidae) eggs, which are known to represent suitable hosts for the development of this parasitoid species in the laboratory.

## Materials and Methods

### Insects

Colonies of the three species of insects used in the experiments were kept in the laboratory in a room under controlled conditions at a temperature of 25 ± 1 °C, relative humidity (RH) of 70 ± 10%, and photophase of 12 h of light and 12 h of darkness. *C*. *cephalonica* eggs were obtained from Embrapa Soja of Londrina, PR, Brazil, and kept in the laboratory since 2014 following the methodology described by Bernardi *et al*.^17^. *H*. *armigera* individuals were obtained from soybean crops in Luís Eduardo Magalhães, BA, Brazil (12°5′58″S, 45°47′54″W), and were reared in the laboratory for five generations following the methodology described by Abbasi *et al*.^18^. *T*. *pretiosum* individuals were obtained from BUG Agentes Biologicos, Piracicaba, SP, Brazil, and reared in the laboratory following the methodology described by Parra^19^. The *T*. *pretiosum* colony was composed only of females, which reproduced by thelytokous parthenogenesis^20^.

### Parasitism by *T*. *pretiosum* on *H*. *armigera* eggs

Eggs were used that were up to 24 h of age (i.e. up to 24 h after female oviposition), from the first day of female oviposition on *C*. *cephalonica* (alternative host) and *H*. *armigera* (natural host). The egg volume of *H*. *armigera* is 0.08 mm^3 ^^21^, and that of *C*. *cephalonica* is 0.036 mm^3 ^^22^. The eggs were glued to light blue paper (3.5 × 1.5 cm) with arabic gum (50%) diluted in deionized water. *C*. *cephalonica* eggs were handled with a soft-bristled brush, while those of *H*. *armigera* were cut out individually from the paper that was used as an oviposition substrate and glued to the light blue paper. Both types of eggs were exposed to germicidal light for 45 min to arrest their development. Each experimental replicate corresponded to a flat-bottomed glass tube (8.0 cm high × 2.0 cm in diameter), which contained a piece of light blue paper with eggs and a female parasitoid, and was covered with polyvinyl chloride plastic film. A droplet of honey was placed on the inner surfaces of the tubes to feed the females. Twenty replicates were used, with 30 eggs per host. Two sets of females were used in different treatments, one in which the female was allowed to parasitize the eggs for 24 h, while in the other treatment it was allowed to parasitize the eggs for 48 h, after which they were withdrawn and discarded. Female wasps used in the experiments were up to 24-h-old. The tubes were subsequently checked for adult emergence once per day. Parasitism frequency was evaluated by counting the number of dark eggs (signaling the occurrence of parasitism), and the emergence rate was determined by counting the number of dark eggs with holes. The duration of the period elapsed from the egg to adult parasitoid was also determined, and was measured from the day of parasitism to the day of offspring emergence. Laboratory conditions were controlled at 25 ± 2 °C with a 70 ± 10% RH and a 12-h photoperiod during these experiments.

### Longevity of *T*. *pretiosum*

After the emergence of the parasitoids in response to the 24-h treatment described in the previous section (“Parasitism by *Trichogramma* on *H*. *armigera* eggs”), 40 females from both hosts were randomly selected and used in further experiments to determine the longevity of the adults. The females were kept individually in flat-bottomed glass tubes (8.0 cm high × 2.0 cm in diameter), which contained a honey droplet on their inner surface for food and were covered with polyvinyl chloride plastic film. Each wasp was provided honey *ad libitum*, and thus food level did not affect how long each wasp lived. Laboratory conditions were controlled at 25 ± 2 °C with a 70 ± 10% RH and a 12-h photoperiod.

### Parasitism of *T*. *pretiosum* on eggs oviposited by *H*. *armigera* on different days

Eggs from the first, second, third, fourth, fifth, and sixth day of *C*. *cephalonica* and *H*. *armigera* oviposition were used in this test. Thirty eggs from each host collected on different days of oviposition were exposed to a *T*. *pretiosum* female for 24 h in a flat-bottomed glass tube (8.0 cm height × 2.0 cm in diameter) containing a droplet of honey on the inner-side surface and covered with polyvinyl chloride plastic film. The eggs used in the different treatments were at the same developmental stage (<24 h post-oviposition). The eggs were attached to light blue paper and placed under germicidal light for 45 min, as described in a previous section (“Parasitism by *Trichogramma* on *H*. *armigera* eggs”). Fifteen replicate tests were performed, with 30 eggs used for each host, in which parasitism and parasitoid emergence frequencies were evaluated. Female wasps used in the experiment were up to 24-h-old. Laboratory conditions were controlled at 25 ± 2 °C with a 70 ± 10% RH and a 12-h photoperiod.

### Parasitism by *T*. *pretiosum* on eggs of *H*. *armigera* of different ages

Each female parasitoid was placed in a glass tube (8.0 cm height × 2.0 cm in diameter) covered with polyvinyl chloride plastic film and with a honey droplet provided on the inner wall for food. Thirty *C*. *cephalonica* eggs that were 1-, 2-, 3-, or 4-days-old were obtained from laboratory rearing, glued onto light blue paper (3.5 × 1.5 cm), and then placed into the glass tubes. This procedure was repeated with *H*. *armigera* eggs. The eggs used in this experiment were not placed under germicidal light because in this experiment we wanted to study the development of the embryo after parasitism. Female wasps used in the experiment were up to 24-h-old. After 24 h of parasitism, *T*. *pretiosum* females were removed and discarded. Fifteen replicates were observed, with 30 eggs for each host. The tubes containing light blue paper were kept in a heated room, under the conditions described previously, until the offspring emerged. The percentages of eggs that were parasitized and underwent parasitoid emergence were also evaluated. Laboratory conditions were controlled at 25 ± 2 °C with a 70 ± 10% RH and a 12-h photoperiod.

### Host preference of *T*. *pretiosum*

**Figure 1:**
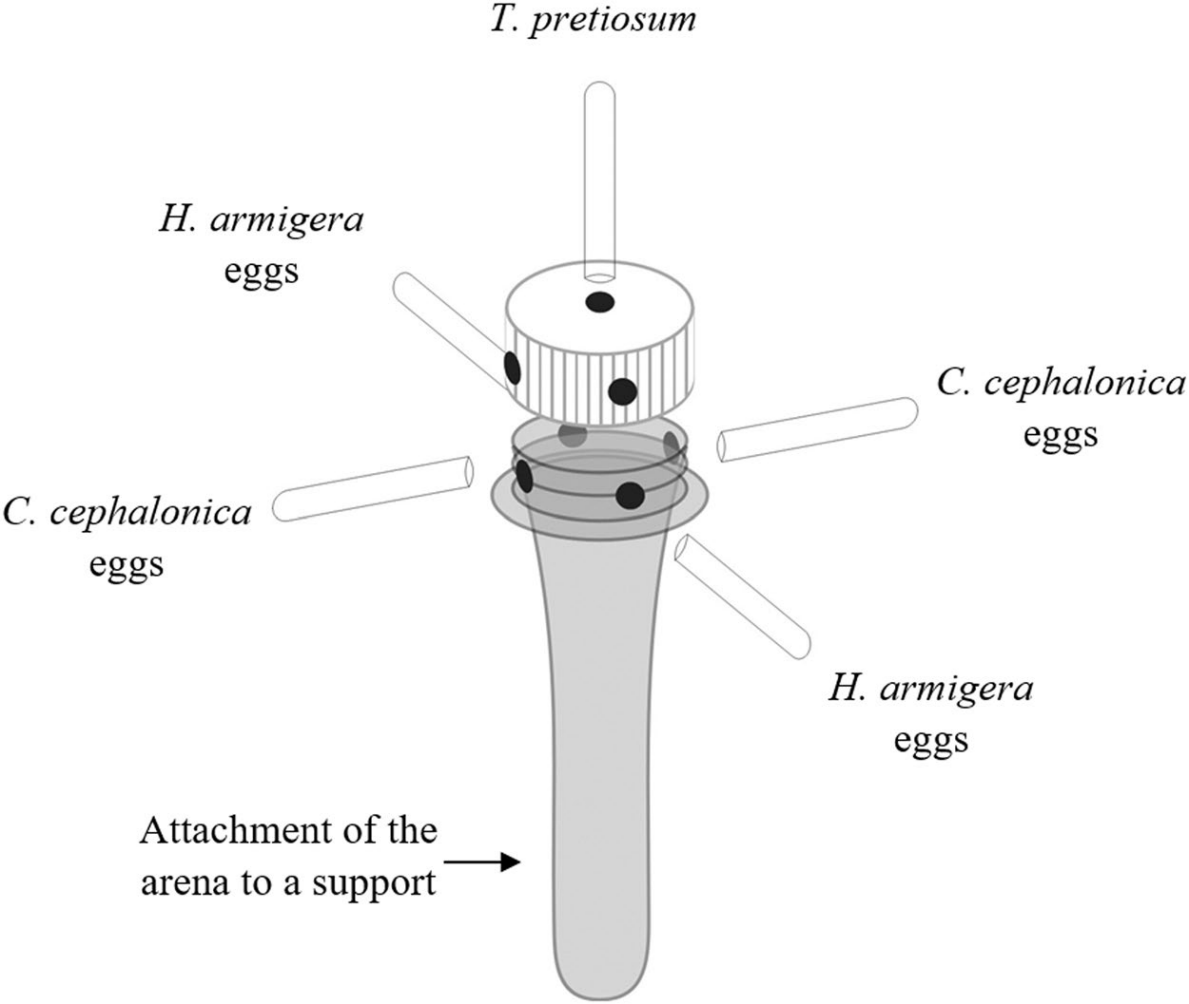
Diagram of the choice test arena used to test the host preference of *Trichogramma pretiosum*.

Arenas were set up that were 4-cm high, made of transparent polyethylene acrylic, and contained four Duran tubes arranged equidistantly from the covering holes (Fig. 1)^23,24^. The insects could move between the different eggs in each tube to choose between them based on touch (egg-size measuring) and odor. In the test with a double chance of two choices, light blue pieces of paper (0.4 × 2.0 cm) containing 15 *C*. *cephalonica* eggs each were placed in two opposing tubes, and 15 *H*. *armigera* eggs were placed in the other two tubes. In the no-chance tests, only two tubes containing 15 eggs of either *C*. *cephalonica* or *H*. *armigera* were placed in the arena. The eggs were placed under germicidal light for 45 min before being used in the test. A female was released into each arena, through a hole located in the top part of the cap. After 24 h, the Duran tubes with the eggs were removed from the arena, covered with polyvinyl chloride plastic film, and kept in a heated room until the adults emerged. Fifteen replicates were observed for each treatment, and the frequencies of parasitism and emergence of offspring were evaluated. Laboratory conditions were controlled at 25 ± 2 °C with a 70 ± 10% RH and a 12-h photoperiod.

### Data analyses

Data on the parasitism, emergence, and egg-to-adult period of *T*. *pretiosum* were submitted to the Kolmogorov and Bartlett tests to verify the normality of their residuals and the homogeneity of their variances, respectively. The data that met these assumptions were then submitted to analysis of variance (ANOVA). When there were two treatments, the Student’s *t-*test was used to compare different treatment conditions to each other, and when there were more than two treatments the Student-Newman-Keuls test was used (P < 0.05). When the data did not meet the requirements for ANOVA, the most adequate transformation was used, and if they still did not present normal residuals and homogeneous variances the data were then submitted to non-parametric tests. For the non-parametric tests, the Wilcoxon test was used to compare two treatments and the Kruskal–Wallis test was used to compare three or more treatments (*p* < 0.05). All analyses were conducted using the SAS software^25^.

Survival curves were also constructed using survival data at specific ages, and were compared according to the Kaplan-Meyer methodology^26^ and analyzed using SAS software^25^.

The frequency data from the choice tests were analyzed using Proc FREQ^25^ and interpreted by the chi-square (χ^2^) test, in which 1:1 was the null hypothesis assumed if the parasitoid had no preference for one host over the other.

## Results

### Parasitism by *T*. *pretiosum* on eggs of *H*. *armigera*

Comparing parasitism on the same host over time, an increase of 27.2% was observed when the female *T*. *pretiosum* was exposed to eggs of *C*. *cephalonica* for 48 h compared to that after 24 h (*t* = −4.90, df = 43, *p* < 0.0001). For *H*. *armigera*, similar levels of parasitism were observed after 24 and 48 h of exposure (*t* = −0.92, df = 43, *p* = 0.3603). When the hosts were compared within the same exposure periods, there was 14.4 and 34.9% greater percent parasitism on eggs of the alternative host *C*. *cephalonica* compared to that on *H*. *armigera* following 24 h (*t* = 2.69, df = 38, *p* = 0.0106) and 48 h (*t* = 5, df = 48, *p* < 0.0001) of parasitoid exposure, respectively (Table 1).

**Table 1:** Percent parasitism of *T*. *pretiosum* on *C*. *cephalonica* and *H*. *armigera* eggs after 24 or 48 h of exposure.

	*C. cephalonica*	*H. armigera*
24 h	57.4 ± 4.65 Ab^a^	43.0 ± 2.65 Ba
48 h	84.6 ± 3.30 Aa	49.7 ± 6.14 Ba

^a^Means ± SE followed by the same letter (capital letters within rows, lowercase letters within columns) did not differ significantly according to Student’s *t*-test (*p* > 0.05).

The duration of the egg-to-adult period of *T*. *pretiosum* was 10 days on both hosts after 24 h of egg exposure. At 48 h, the duration of the developmental period was longer on *C*. *cephalonica* eggs (11.3 days) than on *H*. *armigera* eggs (10.9 days) (z = 2.36, df = 1, *p* = 0.0182) (Table 2).

**Table 2:** Duration of the egg-to-adult period of *T*. *pretiosum* on eggs of *C*. *cephalonica* and *H*. *armigera* after 24 or 48 h of exposure.

	*C. cephalonica*	*H. armigera*
24 h	10.0 ± 0.00 Aa^a^	10.0 ± 0.00 Aa
48 h	1.3 ± 0.09 Ba	10.9 ± 0.12 Aa

^a^Means ± SE followed by the same letter (capital letters within rows, lowercase letters within columns) did not differ significantly according to Student’s *t*-test (*p* > 0.05).

The emergence of parasitoids was not affected by the different hosts following 24 h of exposure (*t* = 0.82, df = 37, *p* = 0.4165). However, after 48 h of exposure percent emergence was 29.2% higher on *C*. *cephalonica* eggs than on *H*. *armigera* eggs (*t* = 5.62, df = 48, *p* < 0.0001). For both hosts, there was a significant difference in the percent emergence between exposure times. The emergence rates on *C*. *cephalonica* (*t* = 4.28, df = 43, *p* = 0.0001) and *H*. *armigera* eggs (*t* = 9.08, df = 43, *p* < 0.0001) were increased 1.2- and 1.86-fold after 24 h (Table 3).

**Table 3:** Percent emergence of *T*. *pretiosum* from eggs of *C*. *cephalonica* and *H*. *armigera* after 24 or 48 h of exposure.

	*C. cephalonica*	*H. armigera*
24 h	96.2 ± 1.37 Aa^a^	94.5 ± 1.65 Aa
48 h	80.1 ± 3.21 Ab	50.9 ± 5.39 Bb

^a^Means ± SE followed by the same letter (capital letters within rows, lowercase letters within columns) did not differ significantly according to Student’s *t*-test (*p* > 0.05).

### Longevity of *T*. *pretiosum*

**Figure 2:**
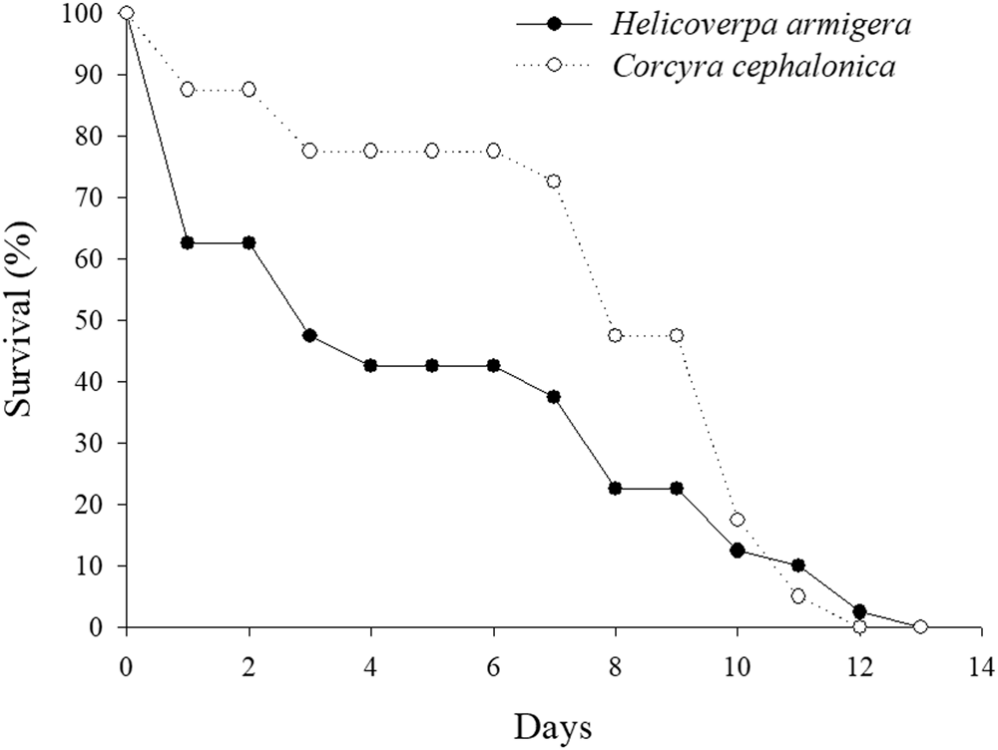
Longevity of *Trichogramma pretiosum* after emergence from *Helicoverpa armigera* and *Corcyra cephalonica* eggs. The treatments differed significantly based on the Wilcoxon test (χ^2^ = 7.52, df = 1, *p* = 0.0061).

Following emergence in host eggs parasitized for 24 h, *T*. *pretiosum* females from *C*. *cephalonica* had a longevity of up to 12 days, which differed in relation to those from *H*. *armigera*, who had a longevity of up to 13 days. However, 3 days after emergence the percentage of surviving adults that had emerged from *H*. *armigera* eggs was reduced to 50%. The equivalent reduction was observed only on day 8 for insects that had emerged from *C*. *cephalonica* eggs. The survival of insects from *C*. *cephalonica* eggs was lower than that of those from *H*. *armigera* eggs on day 11 only (Fig. 2).

### Parasitism of *T*. *pretiosum* on eggs obtained on different days of *H*. *armigera* oviposition

For *C*. *cephalonica* (χ^2^ = 11.89, df = 5, *p* = 0.0362), the parasitism frequency of *T*. *pretiosum* was similar for eggs obtained on different days of oviposition, with the exception of the first day, on which parasitism was 20.2% less frequent than that on the second day. On eggs of *H*. *armigera* (χ^2^ = 34.03, df = 5, *p* < 0.0001), the third day of oviposition presented the highest percent parasitism (71.1%) (Table 4).

**Table 4:** Parasitism (%) of *T*. *pretiosum* on eggs oviposited on different days.

Oviposition period	Host	
	*C*. *cephalonica*	*H*. *armigera*
First day	56.0 ± 3.64 b*^a^	41.1 ± 5.78 c
Second day	76.2 ± 3.09 a*	61.5 ± 3.04 b
Third day	65.6 ± 5.05 ab	71.1 ± 2.02 a
Fourth day	64.1 ± 5.09 ab*	49.8 ± 3.48 c
Fifth day	63.3 ± 6.10 ab	51.6 ± 5.30 bc
Sixth day	68.5 ± 5.85 ab*	38.9 ± 3.71 c

^a^Means ± SE in the same column followed by the same letter did not significantly differ according to the Kruskal–Wallis test (*p* > 0.05); * indicates a significant difference between values in the same row based on Student’s *t*-test (*p* < 0.05).

On the first (*t* = 2.18, df = 28, *p* = 0.0375), second (*t* = 3.40, df = 28, *p* = 0.0021), fourth (*t* = 2.32, df = 28: *p* = 0.0281), and sixth (*t* = 4.50, df = 23, *p* = 0.0002) days of oviposition, the percent parasitism of *C*. *cephalonica* eggs was higher than that on *H*. *armigera* eggs (Table 4).

The percent emergence ranged from 88.7 to 97.6%, and was similar between oviposition periods for *C*. *cephalonica* (χ^2^ = 8.71, df = 5, *p* = 0.1211) and *H*. *armigera* (χ^2^ = 8.28, df = 5, *p* = 0.1414), as well as between these hosts.

### Parasitism of *T*. *pretiosum* on eggs of *H*. *armigera* of different ages

The percent parasitism of *T*. *pretiosum* was lower on 2-day-old *C*. *cephalonica* eggs (χ^2^ = 16.30, df = 3, *p* = 0.0010, 63.3%) and 3-day-old *H*. *armigera* eggs (χ^2^ = 19.99, df = 3, *p* = 0.0002, 41.3%) than on host eggs of other ages (Table 5).

**Table 5:** Parasitism (%) of *T*. *pretiosum* on host species’ eggs of different ages.

Age of the egg	Host	
	*C*. *cephalonica*	*H*. *armigera*
One day	76.7 ± 2.53 a*^a^	61.7 ± 3.04 a
Two days	63.3 ± 2.62 b	65.3 ± 2.58 a
Three days	78.9 ± 1.68 a*	41.3 ± 3.09 b
Four days	68.4 ± 4.73 ab	55.9 ± 4.91 a

^a^Means ± SE in the same column followed by the same letter did not significantly differ according to the Kruskal–Wallis test (*p* > 0.05); * indicates a significant difference between the values in the same row based on Student’s *t*-test (*p* < 0.05).

One- and 3-day-old eggs (*t* = 3.78, df = 28, *p* = 0.0008) presented 15 and 37.6% higher percent parasitism, respectively, on *C*. *cephalonica* than on *H*. *armigera* (Table 5).

The percent emergence was not influenced by the age of eggs in either *C*. *cephalonica* (χ^2^ = 0.07, df = 3, *p* = 0.9949) or *H*. *armigera* (χ^2^ = 3.25, df = 3, *p* = 0.3545), and ranged from 91.1 to 96.4%.

### Host preference of *T*. *pretiosum*

**Figure 3:**
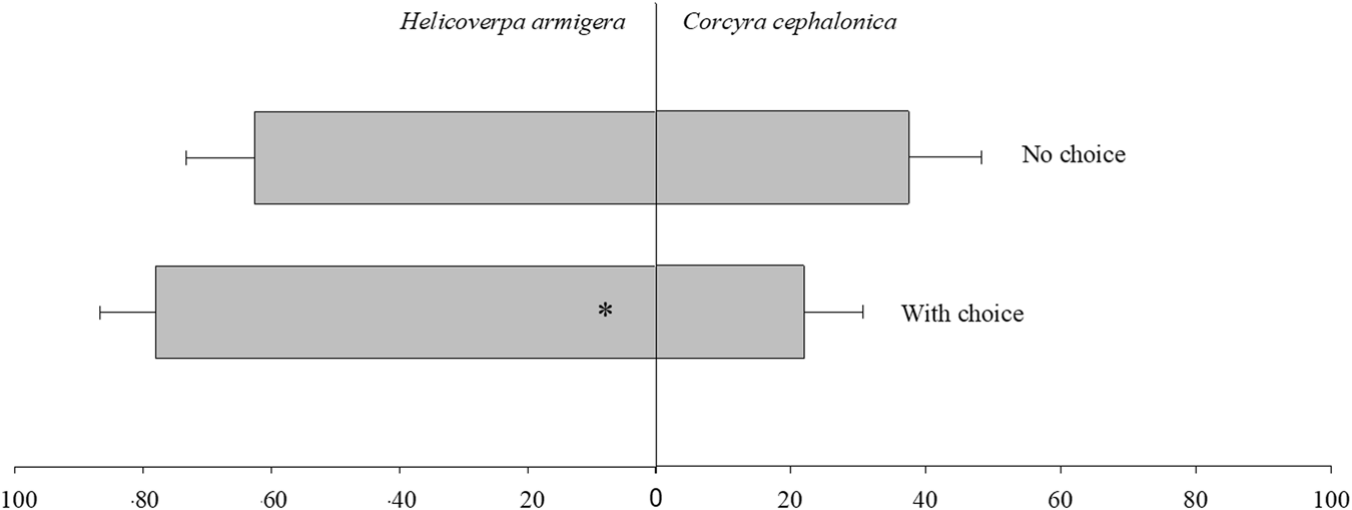
Parasitism (%) of *T*. *pretiosum* on *H*. *armigera* or *C*. *cephalonica* eggs with and without a chance of choice between host species.

In the test where the parasitoid was given a chance of choice between hosts (χ^2^ = 20.12, df = 1, *p* < 0.0001), *T*. *pretiosum* preferred to parasitize *H*. *armigera* eggs (80%) over those of *C*. *cephalonica* (20%). In the tests with no chance of choice (χ^2^ = 1.1843, df = 1, *p* = 0.2765), there was no preference of the parasitoid for one host over the other (Fig. 3).

There was no difference between the hosts in the emergence of adults observed; in the tests with a chance of choice (χ^2^ = 0.0179, df = 1, *p* = 0.8937) and no chance of choice (χ^2^ = 1.2678, df = 1, *p* = 0.2602), *T*. *pretiosum* grew equally well on both *C*. *cephalonica* and *H*. *armigera* eggs.

## Discussion

The present study contributes basic information useful for studies aiming to achieve the management of an introduced pest in Brazil, *H*. *armigera*, using the egg parasitoid *T*. *pretiosum*, which is efficient at controlling some lepidopteran pests in several crops of economic importance^16,27^.

The parasitism frequency of *T*. *pretiosum* over 24 and 48 h of exposure to *C*. *cephalonica* and *H*. *armigera* eggs was higher in the alternative host, *C*. *cephalonica*, which is used to maintain laboratory rearing cultures of the parasitoid. The percent parasitism at 24 and 48 h on *H*. *armigera* eggs was lower than that reported by Ballal and Singh^28^, which was 76.7% under laboratory conditions when using an Indian population of the same pest. However, before tests were done the authors of the present study reared the parasitoid for two generations on *H*. *armigera* eggs. Therefore, it is possible that the better performance of the parasitoid on *C*. *cephalonica* eggs was related to behavioral adaptation (i.e. to insect conditioning), as it was maintained for successive generations in the laboratory on the eggs of this host^29–31^. In the laboratory, insects can adapt their behavior to the conditions in which they are maintained, both in adults and in the young^29^. However, previous results showed that the measures of laboratory performance used (fecundity and offspring sex ratio) were good predictors of field success in *T*. *pretiosum*^32^.

However, the parasitism frequency on *H*. *armigera* eggs observed herein was approximately 50% (49.7%), which suggests that the release of *T*. *pretiosum* in the field would need to be combined with other tools to control the pest effectively. *T*. *pretiosum* can be released in conjunction with the application of chemical and biological products, which must be efficient for use in the control of the pest but also selectively harmless to the parasitoid^33,34^. In sweetcorn crops in eastern Australia, *Trichogramma* release was conducted along with the use of bacteria- and virus-based biological products in IPM for the control of *H*. *armigera*^35^. In China, *Trichogramma* species are also used for pest management in maize, including the control of the eggs of *H*. *armigera*^36^.

In Turkey, the release of 120,000 *Trichogramma* parasitoids per hectare to control *H*. *armigera* on cotton crops resulted in parasitism frequency being reduced to 52.5% of its initial value^37^, highlighting the potential of *Trichogramma* for use in the biological control of *H*. *armigera*.

The percent emergence was similar between the hosts tested following 24 h of exposure, which suggests that the eggs of both species permit the complete development of the parasitoid^38^. However, with 48 h of exposure the emergence rate was reduced in both species to values below the ideal value of 85%^10^. It is possible that by remaining in contact with the eggs for a longer period, some females may deposit their eggs onto a host that is already parasitized, either by itself or by another female, which results in superparasitism. This behavior may impair the development of the parasitoid within the egg, or even kill both the developing parasitoid and the host. When a host is superparasitized, some or all of the immature parasitoids are insufficiently nourished, and thus fail to fully develop and die. In some cases, insects are born with a smaller size or with deformations^39–41^. Superparasitism may therefore reduce the parasitoid’s reproductive success^42^. Furthermore, in larger hosts gregarious development may occur, as is the case for *H*. *armigera* eggs and could explain the apparently reduced levels of parasitism on this species^43^.

In practice, our results regarding exposure time differences suggest that there is a need for periodic releases of the parasitoid, since the parasitoid will not maintain its population at the necessary level required in the field to control the pest once emergence has declined.

EMBRAPA (the Brazilian Agricultural Research Corporation) recommends that the parasitoid is released when, during sampling with adhesive pheromone traps, the first adults of *H*. *armigera* are collected^44^. However, in this study the highest frequencies of parasitism occurred on eggs collected on the third day of pest oviposition. These results are of great importance to the release of *T*. *pretiosum* as they will help the parasitoid to be released at the most appropriate time to best contribute to the efficient control of *H*. *armigera* in the field.

Regarding the ages of the parasitized eggs, there was reduced parasitism on 2-day-old *C*. *cephalonica* eggs and 3-day-old *H*. *armigera* eggs compared to that at other ages. The age of host eggs may influence the performance of egg parasitoids used in biological control^45^, since eggs may undergo morphological and physiological changes that may interfere with their acceptance by the female^46,47^. As the host egg is in a transitional stage of development, the parasitoid must kill the embryo and prevent its development, and then subsequently oviposit its eggs^48^.

In 3-day-old *H*. *armigera* eggs, it is possible that the embryo is already developing and occupying most of the total volume of the egg, with a high level of sclerotization, which increases the protection of the host and decreases the amount of food available for the development of a *Trichogramma* larva inside the egg^48^.

In general, it can be assumed that there was parasitism on *H*. *armigera* eggs of all ages, indicating the acceptance of the eggs of this species by the parasitoid, even at older stages. If the environmental conditions are favorable to the parasitoid insect for a longer period in the field, it may continue to parasitize pest eggs of all ages present on the crop, preventing the larvae from hatching.

The age of the egg following parasitism did not affect the emergence of the parasitoid offspring in this study, as was also previously observed with *T*. *pretiosum* on *Mocis latipes* eggs (Guenée) (Lepidoptera: Noctuidae)^49^, *T*. *galloi* Zucchi (Hymenoptera: Trichogrammatidae) on *Diatraea saccharalis* (Fabricius) (Lepidoptera: Crambidae)^50^, *T*. *cacoeciae* Marchal (Hymenoptera: Trichogrammatidae) on *Lobesia botrana* (Denis and Schiffermüler) (Lepidoptera: Tortricidae)^12^, and *T*. *Chilonis* Ishii (Hymenoptera: Trichogrammatidae) on *Plutella xylostella* (Linnaeus) (Lepidoptera: Plutellidae)^51^. Therefore, the offspring and their descendants should be able to establish persistent populations in the areas in which they are released.

In the preference tests conducted, when a chance of choice between host species was provided to the parasitoid, it preferred the eggs of the natural host, *H*. *armigera*. Several factors influence host preference, such as egg size, which is a critical factor in host selection. In general, most *Trichogramma* species tend to prefer to oviposit on medium-to large-sized eggs^52^. In the present study, the eggs of *H*. *armigera* and *C*. *cephalonica* had an approximate volume of 0.08 mm^3^ and 0.036 mm^3^, respectively^21,22^, which may be one of the factors explaining this preference. Further studies on the mechanisms associated with host selection should be performed with *T*. *pretiosum* and *H*. *armigera* to guarantee the success of the application of this parasitoid in the biological control of the pest.

The results obtained in this study demonstrated the possibility of using *T*. *pretiosum* in the control of *H*. *armigera*. Semi-field and field tests should be performed to adjust and optimize the release methodology, as well as studies of the association of this parasitoid with other pest control techniques.
